# Fatty Acids and Eicosanoids Change during High-Fiber Diet in NAFLD Patients—Randomized Control Trials (RCT)

**DOI:** 10.3390/nu14204310

**Published:** 2022-10-15

**Authors:** Dominika Maciejewska-Markiewicz, Arleta Drozd, Joanna Palma, Karina Ryterska, Viktoria Hawryłkowicz, Patrycja Załęska, Ewa Wunsh, Katarzyna Kozłowska-Petriczko, Ewa Stachowska

**Affiliations:** 1 Department of Human Nutrition and Metabolomics, Pomeranian Medical University in Szczecin, Broniewskiego 24, 71-460 Szczecin, Poland; 2 Department of Biochemical Science, Pomeranian Medical University in Szczecin, Broniewskiego 24, 71-460 Szczecin, Poland; 3 Translational Medicine Group, Pomeranian Medical University in Szczecin, 70-204 Szczecin, Poland

**Keywords:** fiber, fatty acids, eicosanoids, NAFLD, SCFA

## Abstract

Background: Non-alcoholic fatty liver disease (NAFLD) is a wide spectrum condition characterized by excessive liver fat accumulation in people who do not abuse alcohol. There is no effective medical treatment for NAFLD; therefore, most important recommendations to reduce liver steatosis are diet and lifestyle, including proper physical activity. The aim of our study was to analyze the fatty acids and eicosanoids changes in the serum of patients who consumed high-fiber rolls for 8 weeks. Materials and Methods: The group of 28 Caucasian participants was randomly divided into two groups, those who received 24 g of fiber/day—from 2 buns of 12 g each (n = 14), and those who received 12 g of fiber/day—from 2 buns of 6 g (n = 14). At the beginning and on the last visit of the 8-week intervention, all patients underwent NAFLD evaluation, biochemical parameter measurements, and fatty acids and eicosanoids evaluation. Results: Patients who received 12 g of fiber had significantly reduced liver steatosis and body mass index. In the group who received 24 g of fiber/day, we observed a trend to liver steatosis reduction (*p* = 0.07) and significant decrease in aspartate aminotransferase (*p* = 0.03) and total cholesterol (*p* = 0.03). All changes in fatty acid and eicosanoids profile were similar. Fatty acids analysis revealed that extra fiber intake was associated with a significant increase in monounsaturated fatty acids and decrease in saturated fatty acids. Moreover, both groups showed increased concentration of gamma linoleic acid and docosahexaenoic acid. We also observed reduction in prostaglandin E_2_. Conclusions: Our study revealed that a high amount of fiber in the diet is associated with a reduction in fatty liver, although this effect was more pronounced in patients in the lower fiber group. However, regardless of the amount of fiber consumed, we observed significant changes in the profile of FAs, which may reflect the positive changes in the lipids liver metabolism. Regardless of the amount of fiber consumed, patients decreased the amount of PGE_2_, which may indicate the lack of disease progression associated with the development of inflammation.

## 1. Introduction

Non-alcoholic fatty liver disease (NAFLD) is a wide spectrum condition characterized by excessive liver fat accumulation in people who do not abuse alcohol [1]. NAFLD can range from simply fat accumulation in the liver (more than 5% of liver weight) by inflammation to fibrosis and cirrhosis, which are the causes of liver failure [2]. NAFLD is also a part of metabolic syndrome, which increases the risk of other metabolic diseases. The prevalence of the disease is estimated at 30% among US adults and is an emerging health problem worldwide [3]. The most effective treatment of NAFLD is lifestyle changes, including proper physical activity and diet reduction [4].

Recent studies indicate that one of the most effective treatments for NAFLD is weight reduction achieved by caloric restriction [5]. Fiber consumption reduces appetite and the frequency of eating by the indirect regulation of orexigenic hormone—ghrelin. Proper amount of fiber intake, along with a low-energy diet, is linked with weight loss and NAFLD regression [6]. The results of a systematic review meta-analysis focused on the relationship between prebiotic supplementation and anthropometric and biochemical parameters in NAFLD patients showed that fiber supplements can improve anthropometric, metabolic, and liver-related biomarkers such as body mass index (BMI), insulin, homeostasis model assessment for insulin resistance (HOMA-IR), aspartate aminotransferase (AST), and alanine aminotransferase (ALT) [7]. Regression of NAFLD reflects in metabolic pathways, which is associated with fatty acids and their derivatives changes [8]. Our research team discovered that reduction in liver steatosis is connected with a significant increase in eicosapentaenoic acid (EPA), docosapentaenoic acid (DPA), docosahexaenoic acid (DHA) and a decrease in palmitoleic acid [9]. Moreover, it seems that fatty acids profile in blood is a good predictor of liver changes, and the potential markers of liver steatosis are oleic acid, vaccenic acid, EPA, DHA, and DPA [10]. The fatty acid derivatives (eicosanoids) responsible for the initiation/reduction of inflammation are also associated with various NAFLD stages [11]. The most promising markers of steatosis progression are high-inflammatory cytokines, 9-hydroxyloctadecadienoic acids (9-HODE), and 13-hydroxyloctadecadienoic acids (13-HODE) [12]. The aim of our study was to analyze the fatty acids and eicosanoids profiles in the serum of patients who consumed high-fiber rolls for 8 weeks.

## 2. Materials and Methods

### 2.1. Design of the Study

The study (ClinicalTrials.gov Identifier: NCT04520724) was conducted in 2019, between July and November. All participants were enrolled to the project from the Sonomed Medical Centre in Szczecin, Poland. Inclusion criteria: FibroScan^®^ (CAP > 234 dB/m), age > 18 years. Exclusion criteria: infection with HBV (Hepatitis B Virus), HCV (Hepatitis C Virus), HAV (Hepatitis A Virus), body mass index (BMI) > 35 kg/m^2^, changes in physical activity during the study, alcohol consumption (>30 g in men and 20 g in women per day), autoimmune disease, drug use, probiotic supplementation, NSAIDs use (14 days before and during the study), common infection (e.g., cold). Patients underwent 3 visits, first at the baseline, second after 30 days, and third after 60 days. At the baseline and in the last control, FibroScan^®^ was performed. A group of 28 Caucasian participants were included, and 58% were men. Patients were randomly divided into two groups: those who received 24 g of fiber—from 2 buns per 12 g (n = 14), and those who received 12 g of fiber—from 2 buns per 6 g (n = 14). In all visits, biochemical parameters and blood for serum were collected. All biochemical procedures were performed at the laboratory in the Independent Public Regional Hospital in Szczecin. The study protocol was approved by the ethics committee of the Pomeranian Medical University (Szczecin, Poland, KB-0012/131/19) and conformed to the ethical guidelines of the 1975 Declaration of Helsinki. The volunteers provided written informed consent before the study. There was no difference in age, gender distribution, or biochemical parameters at baseline between groups.

### 2.2. Dietary Guidelines for Patients

All patients underwent a 30-min conversation with a licensed nutritionist about the principles of the Mediterranean diet, according to the Mediterranean Diet Foundation. Moreover, all participants were instructed to eat two high-fiber buns (6 g or 12 g per buns) each day during the study. No specific changes in diet (excluded the high-fiber buns) and physical activity were performed during the study.

### 2.3. Fatty Acids (FAs) Analysis

A total 0.5 mL of serum was added to 3 mL of Folch mixture [13] (2:1; v:v; chloroform (Merck KGaA, cat no. 34854): methanol (Merck KGaA, cat no. 1.06018)), 100 μL of Butylated hydroxytoluene (Merck KGaA, cat no. B1378), and 100 μL of internal standard (C21:0 (Merck KGaA, cat no. 51535), 2 mg/mL). Then, the solution was vortexed for 20 min to extract the lipid phase. Next, samples were centrifuged at 15,000 rpm for 15 min. Supernatant was saponified with 1 mL of 2 M KOH (Merck KGaA, cat no. H1758) methanol solution at 70 °C for 20 min and then methylated with 2 mL of a 14% solution of boron trifluoride in methanol (Merck KGaA, cat no. B1252) under the same conditions. A total 2 mL n-hexane (Merck KGaA, cat no. 270504) and 10 mL of saturated NaCl (Merck KGaA, cat no. S3014) solution were added. For gas chromatography analysis (GC), 1 ml of hexane phase was collected. GC was performed using Agilent Technologies 7890A GC System (Agilent (Technologies, Santa Clara, CA, USA) with a SUPELCOWAX 10 Capillary GC Column (15 m × 0.10 mm, 0.10 μm; Supelco, cat no. 24343). The temperature conditions were as follows: initiating temperature 40 °C for 0.5 min; then, increased by 25 °C/min up to 195 °C for 0 min, by 3 °C/min to 205 °C for 0 min, and by 8 °C/min to 250 °C for 0.5 min (total analysis time was 16.158 min). Hydrogen was the carrier gas with gas flow 1 mL/min. FAs were identified by comparing their retention times with standards (Food Industry FAME Mix (cat. no 35077) (Restek)) [14].

### 2.4. Eicosanoids Analysis

Eicosanoids were extracted from the serum using solid-phase extraction RP-18 SPE columns (Agilent Technologies, Wokingham, UK, cat. no 60108-304). A total 0.5 mL of plasma was precipitated with 1 mL of acetonitrile (Merck KGaA, Darmstadt, Germany, cat no. 20060.320). Then, 50 µL of internal standard (Prostaglandin B2, 1 µg/mL (Cayman, cat no. 11210)) was added and samples were incubated for 15 min at −20 °C. Samples were centrifuged at 10,000 rpm for 10 min and the supernatant was collected. An amount of 4.5 mL of 1 mM HCl was added and the pH was adjusted to 3 by adding 1 M HCl. The SPE columns were activated with 3 mL 100% acetonitrile and 3 mL 20% acetonitrile in water. The samples were loaded and double washed with 3 mL 20% acetonitrile in water. Eicosanoids were eluted with 1.5 mL of methanol and ethyl acetate (1:1; v:v), dried under a vacuum, and dissolved in 100 µL of 60% methanol in water with 0.1% acetic acids. Eicosanoids were analyzed using high-performance liquid chromatography (HPLC). The HPLC separations were performed using an Agilent Technologies 1260 liquid chromatograph (Technologies, Santa Clara, CA, USA) with diode array detector (DAD, model G1315CDAD VL+). The separation column was Thermo Scientific Hypersil BDS C18 column (100 × 4.6 mm 2.4 µm, cat no. 28102-154630) with 20 °C of separation temperature. The HPLC condition were as follows: the mobile phase was composed of a mixture of solvent A (methanol: water: acetic acid; 50:50:0.1; v:v:v) and solvent B (methanol: water: acetic acid; 100:0:0.1; v:v:v). Solvent B in the mobile phase was 30% at 0 to 2 min of separation, increased linearly to 80% at 33 min, was 98% between 33.1 and 37.5 min, and was 30% between 40.3 and 45 min, with a flow rate of 1.0 mL/min. The injection volume was 60 µL. The DAD detector monitored peaks by adsorption at 235 nm, 280 nm, and 210 nm [14].

### 2.5. Statistical Analysis

The statistical analysis was performed using the “R 4.0.3” software. The normality of continuous variables distribution by means of Shapiro–Wilk test was evaluated and non-parametric tests were used. Data are presented as medians and interquartile ranges (IQR). Mann–Whitney U test was used to analyze the differences between the groups. In order to estimate the correlation between outcomes of interest, Spearman’s correlation test was used. The values of *p* < 0.05 were considered statistically significant.

## 3. Results

### 3.1. NAFLD and Stiffness Status Evaluation

Patients who received 12 g of fiber showed significantly reduced liver steatosis in FibroScan measurements (CAP 324 dB/m (70 dB/m) vs. CAP 269 dB/m (66.25 dB/m), *p* = 0.02); there was a trend to reduce stiffness status in the liver (VCTE 5.7 kPa (2.17 kPa) vs. VCTE 5 kPa (1.35 kPa, *p* = 0.06). In the group who received 24 g of fiber, we noticed a trend to liver steatosis reduction (CAP 295 dB/m (93 dB/m) vs. CAP 274 dB/m (66 dB/m), *p* = 0.07); the stiffness status did not have a significant change (VCTE 6 kPa (2.4 kPa) vs. VCTE 6 kPa (1.5 kPa), *p* = 0.7).

### 3.2. Biochemical Parameters

Patients who received 12 g of fiber significantly reduced their body weight. In the group where participants received 24 g of fiber, we observed significant reduction of the total cholesterol of total cholesterol and AST. We also noticed a decreasing trend in ALT, LDL, and fasting insulin and an increasing trend in HDL. The results are presented in Table 1.

### 3.3. Fatty Acids and Eicosanoids Analysis

Eight-week high-fiber buns intervention revealed changes in fatty acids and their derivatives in both groups. We can notice a significant reduction in saturated fatty acids and an increase in monounsaturated and polyunsaturated fatty acids. Moreover, the group that received 24 g of extra fiber had increased levels of DHA. Results of fatty acids analysis are presented in Table 2.

## 4. Discussion

Dietary fiber is one of most important components of a healthy diet [15]. Dorosti et al. conducted a randomized, open-label control trial on 112 NAFLD patients, who increased fiber intake in their diet. The results showed that 12 weeks of increased consumption of whole-grain products promoted weight reduction and improved glucose regulation in overweight adults compared with placebo. At the same time, the degree of steatosis had a significant decrease in the intervention group (*p* < 0.001), as well as serum concentration of ALT (*p* < 0.001), AST (*p* < 0.001), γ-glutamyltransferase (GGTP, *p* = 0.009), diastolic blood pressure (*p* = 0.008), and systolic blood pressure (*p* = 0.004) [16]. Javadi et al. showed that prebiotic supplementation (10 g/day for 3 months) in NAFLD patients can significantly reduce the concentration of LDL (*p* = 0.015) and body weight (*p* < 0.001) [17].

Our intervention was based on the additional supply of 12 g and 24 g of fiber. The results of biochemical studies showed that patients who received more fiber (24 g) reduced their total cholesterol and AST concentration. We also noticed a trend to increase HDL, and to decrease LDL, ALT, fasting insulin, and degree of steatosis. Interestingly, the group with less fiber supplementation showed significantly reduced body weight and degree of liver steatosis but we did not see any significant changes in biochemical parameters. However, we can notice a trend to increased fasting insulin in these patients (*p* = 0.09). A number of studies present hypotheses where fiber fermentation products, mainly short-chain fatty acids (SCFAs), contribute additional calorie intake through fermentation, and this can explain weight gain in some individuals [18]. It seems that we can notice this dependence in our patients. SCFAs have a huge impact on glucose and lipid metabolism; therefore, biochemical changes can be more visible in the group of higher fiber intake.

Data from animal studies showed that dietary fiber normalized the expressions of sterol regulatory element-binding protein 1 (SREBP1) [19]. Synthesis of fatty acid in the liver, as well as triglyceride, is regulated by transcription factor SREBP-1c, which increases formation of palmitic acid and its metabolites of elongation and desaturation—stearic acid, palmitoleic acid, and oleic acid. These changes are stimulated by insulin and intracellular cholesterol concentration, which accelerates liver fat accumulation [20,21]. Dietary fiber is fermented by intestinal microbiota, which results in the production of SCFAs. SCFA can regulate many processes involved in glucose and lipid metabolism and reduce endogenous synthesis of cholesterol and fat storage [22]. Recent studies revealed that oral administration of SCFAs tends to downregulate expression of SREBP-1C in liver and fatty acid synthase (FAS), enzymes that induce de novo lipogenesis [22].

Our study showed that increased fiber intake helps to reduce the concentration of main saturated fatty acids, such as palmitic acid and stearic acid, and increase the concentration of monounsaturated fatty acids, such as palmitoleic acid and oleic acid (trend to reduction, *p* = 0.07). These changes are noticeable in both groups. We can assume that increased amount of fiber in the diet reduced de novo lipogenesis, especially because total cholesterol, one the main factors inducing FAS activity, decreased in both groups. In NAFLD patients, adipose tissue delivers approx. 60% FAs for TG synthesized in the liver while de novo lipogenesis contributes only 26% [23]. Enteral infusion of SCFA (acetate and propionate) causes a 40% reduction in serum FAs [24]. The contribution of gut-microbiota-derived acetate production available for whole organism is estimated to be 44% [25]. Increasing fiber fermentation and SCFA production may be a novel strategy to induce regulation of lipid metabolism in liver diseases.

Eicosanoids, especially series 2 of prostaglandins (PGs), play an important role in the development of metabolic diseases and NAFLD. There are three series of homologues PGs, which are biosynthesized from different polyunsaturated fatty acids (PUFA) by cyclooxygenase (COX) [26]. The 2 series PGs are produced from DGLA, an AA derived from membrane phospholipids. PGs have a varied effect on hepatic insulin signaling, dependent on which series of PGs are produced and where they are produced. Metabolites generated in parenchymal hepatocytes or Kupffer cells play a negative role in insulin metabolism [27]. Animal research has revealed that COX-2 inhibitors intake can decrease PGE metabolites in obese rats. These changes were associated with insulin sensitivity improvement and decreasing hepatic glucose production [28].

PGs also participate in lipid metabolism dysregulation and in hepatic lipid storage [29]. PGE_2_ acts with insulin in the pathogenesis and progression of hepatic steatosis. High PGE_2_ concentration in the serum is also associated with fibrosis progression in liver steatosis [30]. The 2 series of PG decreases the secretion of very-low-density lipoprotein (VLDL) and promotes steatosis in hepatocytes. Reduction of VLDL secretion is associated with decreased TG transportation and output [31]. During NAFLD development, increased COX-2 activity and high PGE2 concentration results in lipid storage and peroxidation enhancement in mice [32]. However, some scientific evidence derived from cell culture research suggests that PGE_2_ may play a protective role for liver cells. Our research revealed that, along with the reduction of steatosis, the concentration of PGE also decreased. Moreover, in both groups, we noticed increased concentration of GLA, the precursor of PGs. This observation may indicate the reduction of COX-2 activity in NAFLD patients on a high-fiber diet. The role of fiber and SCFA in COX activity is still unknown.

## 5. Conclusions

NAFLD is associated with metabolic changes connected with lipid metabolism and glucose homeostasis. There are increasing interests about the role of dietary fiber in supporting the treatment of the disease. Our study revealed that a high amount of fiber in the diet is associated with a reduction in fatty liver, although this effect was more pronounced in patients in the lower fiber group, who had reduced body weight. This observation can be caused by higher calorie intake from extra fiber; therefore, calorie restriction and weight loss seems to be a primary goal for NAFLD patients. However, regardless of the amount of fiber consumed, we observed significant changes in the profile of FAs, which may reflect the positive changes in lipid liver metabolism. Regardless of the amount of fiber consumption, patients decreased the amount of PGE_2_, which may indicate the lack of disease progression associated with the development of inflammation.

**Figure 1:**
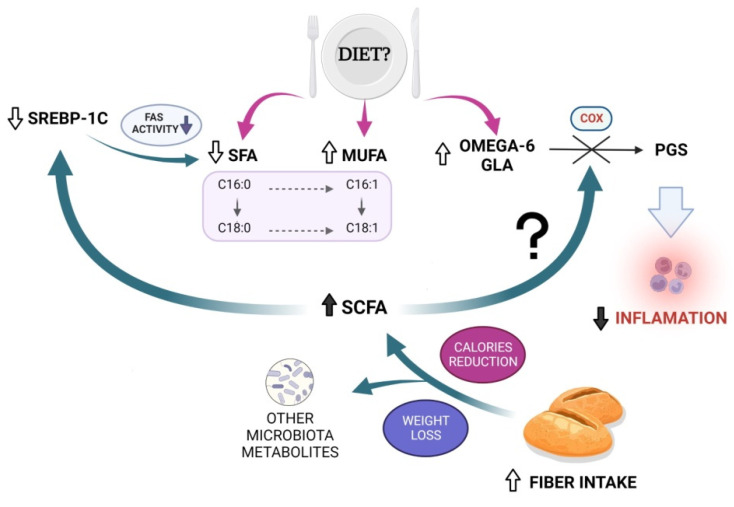
Results of the study and proposed hypothesis.

Figure 1 summarizes results of the study and the proposed hypothesis.

## Figures and Tables

**Table 1:** Differences between biochemical parameters before and after 8 weeks of high-fiber buns intervention.

Parameters	Intervention with 12 g				*p*	Intervention with 24 g				*p*
	Median	IQR	Median	IQR		Median	IQR	Median	IQR	
Fasting glucose (mg/dL)	95	14.1	90	33.5	0.66	91.6	15.4	97.9	10.6	0.94
Total cholesterol (mg/dL)	195.2	53.2	178.2	27.5	0.18	210	51.6	197.3	47.7	0.03 ↓
HDL (mg/dL)	45.2	12	42.8	16.1	0.58	47.1	6.4	48.7	6.5	0.07
LDL (mg/dL)	127.9	57.7	110.4	41.8	0.33	138.9	31.9	129.7	46	0.09
TG (mg/dL)	164.1	95.3	119.7	165	0.14	169.6	152.3	161.6	113.2	0.11
ALT (U/L)	38	19	38	19	0.94	41	21	31	18	0.06
AST (U/L)	26	12	30	11	0.68	26	8	23	6	0.03 ↓
GGTP (U/L)	32	10	35	16	0.23	28	12	24	14	0.12
Fasting insulin (uU/mL)	19.3	20.7	16.9	13.6	0.66	33.7	94.9	37.6	31	0.09
Age (years)	47	12.8	-	-	-	47.5	14.7	-	-	-
BMI (kg/m^2^)	29.1	3.8	28.7	5.2	0.04 ↓	28.2	10.5	28.9	9.7	0.54

↑- significant increase, ↓ significant decrease.

**Table 2:** Fatty acids profile before and after 8 weeks of high-fiber buns intervention.

Fatty Acid (%)	Intervention with 12 g				*p*	Intervention with 24 g				*p*
	Median	IQR	Median	IQR		Median	IQR	Median	IQR	
C13:0 Tridecanoic acid	0.20	0.05	0.20	0.08	0.53	0.22	0.06	0.23	0.09	0.45
C14:0 Myristic acid	1.24	0.67	1.30	0.30	0.35	1.27	0.30	1.11	0.43	0.76
C14:1 Myristolenic acid	0.05	0.06	0.06	0.04	0.0001 ↑	0.05	0.03	0.07	0.04	0.001 ↑
C15:0 Pentadecanoic acid	0.16	0.07	0.19	0.03	0.15	0.14	0.05	0.15	0.05	0.58
C16:0 Palmitic acid	3.46	2.08	29.27	1.89	0.001 ↓	31.45	2.56	29.98	1.86	0.02 ↓
C16:1 Palmitoleic acid	1.03	0.43	1.64	0.99	0.001 ↑	1.15	0.49	1.20	0.90	0.001 ↑
C17:0 Heptadecanoic acid	0.25	0.04	0.26	0.05	0.3	0.24	0.05	0.25	0.07	0.07
C17:1 Heptadecanoic acid	0.06	0.02	0.08	0.03	0.0001 ↑	0.06	0.02	0.07	0.06	0.0001 ↑
C18:0 Stearic acid	21.39	3.98	19.12	3.63	0.03 ↓	21.76	5.53	19.78	5.61	0.019 ↓
C18:1n9 Oleic acid	12.44	4.57	14.50	4.48	0.07	12.04	4.45	14.37	3.67	0.07
C18:1n7 Vaccinic acid	1.34	0.49	1.47	0.41	0.71	1.30	0.42	1.59	0.53	0.17
C18:2n6 Linoleic acid	12.25	5.12	14.07	4.87	0.02 ↑	12.12	5.26	14.67	3.75	0.016 ↑
C18:3n6 Gamma linoleic acid (GLA)	0.17	0.10	0.26	0.16	0.003 ↑	0.14	0.06	0.24	0.10	0.021 ↑
C18:3n3 Linolenic acid	0.18	0.07	0.13	0.06	0.02 ↑	0.17	0.05	0.12	0.04	0.09
C20:4n6 Arachidonic acid (AA)	6.03	1.46	5.76	1.73	0.47	5.75	1.10	6.10	1.44	0.76
C20:5n3 EPA	0.48	0.11	0.51	0.21	0.14	0.58	0.40	0.51	0.55	0.46
C22:4n6 Docosatetraenoic acid	0.49	0.24	0.39	0.29	0.46	0.50	0,19	0.46	0.16	0.32
C22:5n3 Docosapentaenoic acid	0.56	0.18	0.63	0.19	0.05 ↑	0.52	0.18	0.62	0.33	0.008 ↑
C22:6n3 DHA	1.55	0.51	1.50	0.44	0.46	1.60	0.59	1.85	0.90	0.005 ↑

Increased amount of fiber in the diet reduced concentration of prostaglandin E2, which was more visible in the group who received 24 g of fiber (Table 3). ↑- significant increase, ↓ significant decrease.

**Table 3:** Eicosanoids profile before and after 8 weeks of high-fiber buns intervention.

Eicosanoids (ng/mL)	Intervention with 12 g				*p*	Intervention with 24 g				*p*
	Median	IQR	Median	IQR		Median	IQR	Median	IQR	
Resolvine E_1_	0.52	0.26	0.43	0.15	0.1	0.51	0.17	0.33	0.27	0.3
Prostaglandin E_2_	7.42	2.84	5.25	3.78	0.017 ↓	9.89	6.95	5.61	9.51	0.008 ↓
LTX A_4_	3.72	8.93	4.04	6.58	0.89	5.74	3.20	7.92	11.52	0.42
DiHDHA Protectin DX	0.58	0.39	0.13	0.06	0.58	0.33	0.12	0.14	0.05	0.01
Leucotriene B_4_	0.03	0.04	0.03	0.02	0.41	0.04	0.03	0.04	0.01	0.52
18 HEPE	0.11	0.08	0.09	0.05	0.54	0.16	0.07	0.09	0.05	0.3
13 HODE	0.08	0.06	0.10	0.04	0.78	0.09	0.07	0.13	0.11	0.36
9 HODE	0.06	0.03	0.09	0.07	0.27	0.10	0.05	0.12	0.08	0.23
15 HETE	0.63	0.30	0.73	0.62	0.41	0.75	0.47	0.70	0.41	0.63
17 HDHA	0.15	0.09	0.12	0.05	0.68	0.19	0.29	0.12	0.04	0.19
12 HETE	1.45	1.19	1.74	4.98	0.43	1.94	0.62	2.65	1.72	0.11
5 HETE	0.52	0.49	0.79	0.86	0.54	0.67	0.40	0.68	0.70	0.78
5 oxo ETE	0.66	0.67	0.72	0.37	0.21	0.43	0.65	0.62	0.50	0.26

↑- significant increase, ↓ significant decrease.

## Data Availability

Not applicable.

## Abbreviations

NAFLD—Non-alcoholic fatty liver disease; BMI—body mass index; HOMA-IR insulin resistance; AST—aspartate aminotransferase; ALT—alanine aminotransferase; EPA—eicosapentaenoic acid; DPA—docosapentaenoic acid; DHA—docosahexaenoic acid; EPA—eicosapentaenoic acid; DPA—docosapentaenoic acid; GLA—gamma linolenic acid; GGT—γ-glutamyltransferase; HDL—high-density lipoprotein; LDL—low-density lipoprotein; SCFA—short-chain fatty acid; NSAIDs—Non-steroidal anti-inflammatory drugs; PGE_2_—Prostaglandin E_2_; LTX A4—Lipoksyn A4; DiHDHA—dihydroxydocosahexaenoic acid; 18 HEPE—18-hydroxyeicosapentaenoic acid; 13 HODE—13-hydroxyoctadecadienoic acids; 9 HODE—9-hydroxyoctadecadienoic acids; 15 HETE—15-hydroxyeicosatetraenoic acid; 17 HDHA—17-hydroxydocosahexaenoic acid; 12 HETE—12-hydroxyeicosatetraenoic acid; 5 HETE—5-hydroxyeicosatetraenoic acid; 5 oxo ETE—5-oxoeicosatetraenoic acid.
